# Review of Dermoscopy and Reflectance Confocal Microscopy Features of the Mucosal Melanoma

**DOI:** 10.3390/diagnostics11010091

**Published:** 2021-01-08

**Authors:** Andrea De Pascalis, Jean Luc Perrot, Linda Tognetti, Pietro Rubegni, Elisa Cinotti

**Affiliations:** 1 Department of Medical, Surgical and Neurological Science, Dermatology Section, University of Siena, S. Maria alle Scotte Hospital, 53100 Siena, Italy; linda.tognetti@gmail.com (L.T.); pietro.rubegni@gmail.com (P.R.); elisacinotti@gmail.com (E.C.); 2 Department of Dermatology, University Hospital of Saint-Etienne, 42000 Saint-Etienne, France; j.luc.perrot@chu-st-etienne.fr

**Keywords:** melanoma, mucosa, dermoscopy, reflectance confocal microscopy

## Abstract

Mucosal melanoma is a rare tumor with aggressive biological behavior and poor prognosis. Diagnosis is often performed at an advanced stage when the lesions become symptomatic. Although dermoscopy and reflectance confocal microscopy (RCM) are widely used techniques for the diagnosis of cutaneous tumors, their use for mucosal lesions is not well established, probably because the latter are rarer. The objective of this study was to evaluate current literature on these imaging techniques for mucosal melanoma. We searched in PubMed and Cochrane databases all studies up to October 2020 dealing with dermoscopy, RCM, and mucosal melanoma. We found that the most relevant dermoscopic features were structureless pattern and/or the presence of multiple colors. RCM examination mainly showed numerous basal hyper-reflective dendritic cells and loss of normal architecture of the papillae of the lamina propria. Although diagnostic algorithms have been proposed for both techniques, the limit of these methods is the absence of large studies and of standardized and shared diagnostic criteria.

## 1. Introduction

**Figure 1:**
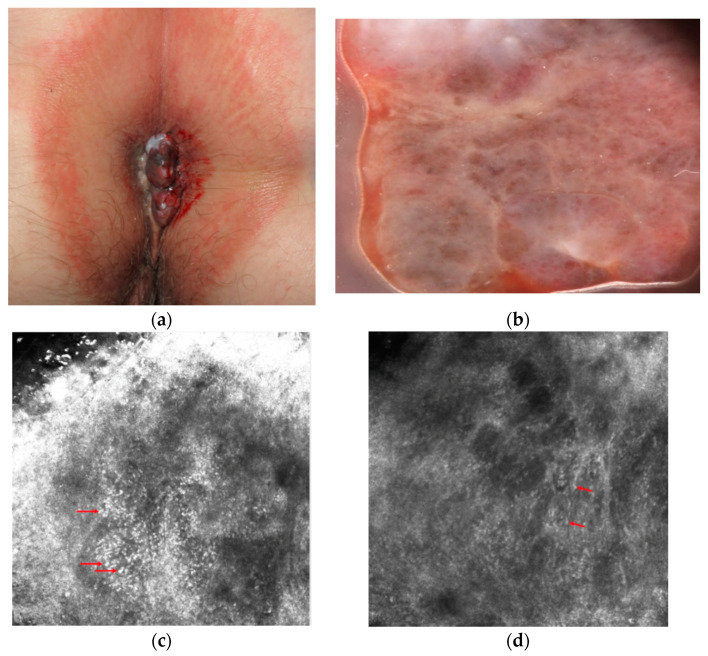
Clinical (**a**), dermoscopic (**b**) and RCM (**c**,**d**) aspect of a hypomelanotic mucosal melanoma. (**b**) Dermoscopy shows structureless grey and white color and remnants of pigmentation. (**c**,**d**) Reflectance confocal microscopy features at the epidermal level (images acquired with VivaScope 3000, Caliber, New York, NY, USA): atypical cells are indicated by red arrows.

Mucosal melanoma can affect the glabrous portion of the lips, oral, sinonasal, genital, urinary, gastrointestinal, anorectal, and conjunctival locations [1]. It is rare, with an incidence rate around 1% of all melanomas [2]. However, because of its anatomic localization and lack of early visible signs and symptoms, it is usually diagnosed at an advanced stage with poor prognosis [3]. Clinically, it most often is a solitary, brown to black macule that can be difficult to differentiate from melanosis that is the most common cause of pigmentation in which can be observed in the mucosa [4,5]. Moreover it is estimated, that about a fifth of mucosal melanomas are amelanotic, which makes the diagnosis even more difficult (Figure 1) [1,6]. In the initial phase of growth, mucosal melanomas are indolent and asymptomatic and most people do not seek medical attention until swelling or ulceration and consequent bleeding occur (Figure 1). 

The histopathological appearance of mucosal melanoma is similar to its cutaneous counterpart. Mucosal melanomas are heterogeneous and can show epitheloid, spindle-shaped, or mixed cytomorphology. This spindle tumor cell type, in particular, is more common in mucosal melanoma than in cutaneous melanoma [7]. The oncogenic drivers of mucosal melanoma are quite different from cutaneous melanoma. The mutation rate of KIT and SF3B1 is higher in mucosal melanoma as compared to cutaneous melanoma. While, the common drivers (BRAF and NRAS) found in cutaneous melanoma have lower mutation rate in mucosal melanoma [1,8].

The biological behavior of mucosal melanoma notoriously differs from cutaneous melanoma. The greater blood and lymphatic flow in mucosa and the differences in the genetic profile of mucosal melanoma eases its local and remote spread. Therefore, mucosal melanoma has a bad prognosis with a five-year survival rate of 10–25% of cases and average survival of two years. If lymphatic glands have been affected, prognosis drops even further [9]. 

Up to date, the only tool available to improve the prognosis of this tumor is its early diagnosis. Dermoscopy and reflectance confocal microscopy (RCM) are broadly used imaging procedures for the non-invasive diagnosis of skin melanoma, but their diagnostic value for mucosal melanoma is not adequately recognized [10,11]. 

Reflectance confocal microscopy (RCM) is a noninvasive technique of skin imaging, which produces high-resolution images of the upper 250 μm of the skin, and is effective in distinguishing between benign and malignant skin lesions [12]. Recently, the RCM devices dedicated to the skin have been also applied to perform “virtual biopsies” of the mucosa [13]. In fact, mucosa is particularly suitable for RCM because of its thin or absent cornified layer and its thin epithelium that allows a deeper penetration of the laser with the consequent possibility of exploring deeper tissue levels. The images offered by RCM correlate with histologic features, with the difference that RCM provides horizontal sections and conventional histology provides vertical sections of the tissue. Moreover, the absence of the stratum corneum determines a higher resolution in the upper layers than in the skin on RCM, having a better-detailed visualization of the cellular morphology [12]. 

The mucosa is similar to the skin with an epithelium corresponding to the epidermis and a lamina propria corresponding to the dermis. Therefore, RCM shows in the epithelium the same honeycomb pattern of the epidermis, characterized by polygonal cells with a hyper-reflective outer part (mainly corresponding to the cellular membrane) and a hypo-reflective inner part (mainly corresponding to the cytoplasm); nuclei are easily visible as bright large round structures in the center of the cells. The lamina propria consists of bright collagen fibers arranged in bundles. Papillae are less evident than in the skin due to the flattened epithelium. When papillae are visible, they are edged and grouped in small clusters with a roundish or elongated shape, thus defining the “ringed” pattern and the “draped” pattern, respectively. Moreover, papillae are rimmed by monomorphous cells, corresponding to the basal epithelial cells that could be slightly more pigmented (and therefore brighter) than the suprabasal cells. Few dendritic bright cells corresponding to Langerhans cells can also be observed in healthy mucosa [14,15].

We realized a review of the literature on dermoscopy and RCM features of mucosal melanoma. Conventional dermoscopy and RCM probes are applicable only to the external mucosa, therefore our research was conducted considering only external part of the oral and genital mucosa (i.e., lip, gum, vulva, penis, and anus). Conjunctival melanoma was excluded from this study due to its peculiar dermoscopy and RCM features [16,17,18].

## 2. Materials and Methods

We searched in PubMed and Cochrane databases for all the articles dealing with dermoscopy and RCM for the diagnosis of melanoma of the oral and genital mucosa up to 30 October 2020. Search terms employed were “dermoscopy and mucosal melanoma”, “dermoscopy and mucous membrane melanoma”, “imaging and mucous membrane melanoma”, “imaging and mucosal melanoma”, “reflectance confocal microscopy and mucosal melanoma”, “reflectance confocal microscopy and mucous membrane melanoma”. To include all relevant studies, the reference list of all articles was checked for any possible article that was ignored by the initial search. Both letters/clinical reports and original papers were included.

No restriction for language was applied. We excluded articles when: (1) they did not deal with the use of non-invasive imaging techniques or the diagnosis of mucosal melanoma; (2) they described lesions in different body areas (i.e., mucosal sites not easily explorable from the outside); (3) they related to mucosal melanoma but did not present any dermoscopic or RCM images or report.

## 3. Results

We found 202 articles: 184 were excluded and 18 were included in our review with a total of 68 lesions. Among the 184 excluded articles, 170 were excluded based on title and abstract and 14 after complete text reading. In our literature review we found mostly case reports or small case series and just three retrospective and observational studies. This could be explained by the rarity of mucosal melanoma. Most descriptions only concern dermoscopy (51 cases), 23 cases have been evaluated with RCM and only six cases with both techniques. Oral mucosa was affected in 22 cases and genital mucosa in 46 cases. Dermoscopic features of mucosal melanoma most frequently described by the authors were: structureless areas (30/51), blue-white veil (26/51), multicomponent pattern (23/51), and multiple colors (16/51). RCM features of mucosal melanoma most frequently described by the authors were: pagetoid infiltration of hyperreflective and polymorphous cells (21/23), atypical pattern of the epithelium (16/23), and disarranged papillae (15/23). We summarized these findings in Table 1 and Table 2.

## 4. Discussion

### 4.1. Dermoscopy of Mucosal Melanomas

Dermoscopy as a non-invasive technique has become a fundamental part in the evaluation of skin lesions, increasing diagnostic accuracy, particularly for the early detection of melanoma [35]. However, while dermoscopy is widely used for the diagnosis of pigmented and nonpigmented lesions of the skin, until recently its applicability on mucosal lesions was not well established. Examination and evaluation of mucosal lesions could be problematic. First, especially in female patients, the lesion may be on a location difficult to examine and patients are embarrassed; second, the dermoscopic findings could be affected by the mucosa stretching due to the examination; and third, the contact probes should be protected in order to prevent infections [36,37].

The first dermoscopy study on mucosal melanoma was conducted by Lin et al. [19] on 8 melanomas included in a series of 40 pigmented lesions on the mucous membrane and mucocutaneous junction from 37 Japanese patients. Lin et al. found that melanomas mainly presented with the multicomponent pattern (six out of eight, 75%) and the homogeneous pattern (two out of eight, 25%). The dermoscopic features most frequently observed in melanomas were asymmetry of structures, multiple colors, blue-white veil and irregular dots or globules (Table 1). Moreover, each lesion was examinated and scored according to the standard dermoscopic algorithms used for hairy skin, including the ABCD rule, CASH algorithm, Menzies method, a three-point checklist, and a seven-point checklist. The majority of the foregoing dermoscopic algorithms demonstrated high sensitivity (62–100%) and specificity (94–100%) in mucosal melanomas, as when they are applied to skin lesions. Based on these data, Lin et al. were the first to suggest applying the algorithms for pigmented lesions of the skin to lesions on the mucocutaneous junction and mucous membrane.

Subsequently, Ronger-Savle et al. [20] focused their study on the dermoscopy of pigmented vulvar lesions, analyzing 68 histopathologically proven cases comprising five melanomas. The dermoscopic patterns observed in melanomas were multicomponent, and irregular-polycircular or irregular-reticular. Further dermoscopic features classically associated to melanoma, like blue-whitish veil, white veil, regression structures and irregular globules were also found. Vessels, when observed, appeared irregular or like milky-red areas. As suggested by Lin et al., Ronger-Savle et al. applied the algorithms for skin melanoma on their series of five vulvar melanomas, including early lesions, but he obtained a low sensitivity (40–80%). Therefore, they proposed an original algorithm for vulvar pigmented lesions derived from multiple correspondence analysis. They gave one or two points for every variable statistically associated with melanoma (multicomponent pattern, irregular vessels, blue-whitish veil, three or more colors, unilateral and unifocal lesion, palpable lesion, white veil, polycircular pattern, and irregular globules) and a total score ≥4 was defined as a threshold for the diagnosis of melanoma. Ronger-Savle et al. applied this algorithm on their series of five melanomas and the sensitivity and specificity were respectively 100% and 94%. Furthermore, when they applied this method to the previously published cases of dermoscopy-studied mucosal melanomas, sensitivity and specificity remained good (100% and 90%, respectively). 

After the retrospective observational studies of Lin et al. and Ronger-Savle et al., the International Dermoscopy Society (IDS) initiated the first multicenter retrospective and observational study about dermoscopy in pigmented mucosal lesions [21]. Blum et al. investigated 140 lesions, comprising 11 melanomas (7.9% of total). On the basis of the univariate analysis conducted, they found two diagnostic models for the identification of pigmented mucosal lesions by dermoscopy: on the report of the first model, the existence of blue, gray, or white color plus the presence of a structureless zone (even though only parts of the lesion were structureless) was considered as suspect. Regarding the diagnosis of melanoma, this model had a sensitivity of 100%, a specificity of 82.2%, a positive predictive value of 32.4%, and a negative predictive value of 100%. In the second model, they rely only on colors: each lesion that presented blue, gray, or white color was considered as suspect, despite the pattern. The sensitivity for melanoma of this model was 100%; the specificity, 64.3%; and the positive predictive and negative values, 19.3% and 100%, respectively. Therefore, from this multicenter study emerged that the observation of blue, gray, or white color is the most effective clue to distinguish between malignant and benign mucosal lesions by dermoscopy. Moreover, the association of at least one of the three colors and the existence of structureless zones had a higher diagnostic precision (Figure 1).

Recently, a multicenter retrospective study was performed by three centers in Italy focusing on the dermoscopic features of thin (with Breslow thickness ≤ 0.5 mm) and in situ vulvar melanoma [22]. The features most frequently observed in their series were: structureless areas (85.7% of cases), grey areas (78.6% of cases), irregular black–brown dots (71.4% of cases) and blue–white structures (71.4% of cases). 

In addition to the three previous studies, only individual case reports can be found in the literature. We selected eight of them [9,23,24,25,26,27,28,29] that met the criteria of our research. The dermoscopic feature common to almost all the lesions reported is the blue-white veil (6/8), followed by the presence of more than two colors (3/8) and irregular vessels (2/6). The structureless pattern is reported in two cases [19,28], while in one case the author reported the presence of homogeneous areas [27].

In our view, an important problem concerns the nomenclature of the observed dermoscopic pattern. As mentioned above, patterns defined as multicomponent, homogeneous and structureless have been frequently reported in mucosal melanomas. Multicomponent (or polymorphous) pattern is defined, according to Ronger-Savle et al., as the presence in the same lesion of multiple patterns, like the homogeneous, reticular and the globular ones combined asymmetrically, whereas Lin et al. did not clearly define it and just stated that it corresponded to the presence of various dermoscopic features. According to Blum et al., structureless could be defined as the lack of any recognizable structure (dots, globules or clods, circles, or lines), despite the color. According to Ronger-Savle et al. and Lin et al.—who preferred the term homogeneous—structureless, could be considered as synonymous for homogeneous, and it referred to absence of classical dermoscopic criteria for melanocytic lesions such as dots and globules, pigment network, and streaks [20]. 

One of the main finding in melanoma was the presence of multiple colors, being blue, gray and white the most frequent. However, the evaluation and discussion of color requires details about polarized or nonpolarized images that are not always given. Ronger-Savle et al. proposed in their article a new algorithm for the early detection of vulvar melanomas. However, according to us and to Ronger-Savle et al. themselves, the validity of this algorithm should be investigated in larger multicenter collaborative studies [20]. A limitation of the studies over mentioned is that most of the studied lesions were clinically detectable and often in an advanced stage (nine in situ melanoma was included in the recent study of Vaccari et al., while no data about melanoma thickness, according Breslow, were given in the previous studies of Bloom et al., Lin et al. and Ronger-Savle et al.); it is therefore not known if the application of these criteria will aid in the detection of early stage mucosal melanoma [24].

### 4.2. RCM of Mucosal Melanomas

The first study on RCM features of mucosal melanoma was performed by Cinotti et al. that evaluated 10 pigmented genital lesions, including two vulvar melanomas [15]. They found atypical cells and loss of the normal chorion papillae architecture. Atypical cells, corresponding to neoplastic melanocytes, were described as large cells with a bright cytoplasm and an often-evident hypo-reflective nucleus; they had a dendritic or a spindle or roundish shape and were often pleomorphic and scattered in the epithelium. They observed that atypical cells and disarranged papillae, that also are two of the main RCM characteristics of skin melanoma, were always found in mucosal melanoma and were never found in melanosis.

One of the largest RCM experiences about mucosal melanoma is that of Derbarbieux et al. [31]. The RCM images of 54 consecutive patients with an oral or genital macular pigmentation were retrospectively evaluated. Histopathological examination confirmed 10 melanomas. Most relevant aspects in their study were: the presence of roundish bright cells, a high density of atypical dendritic cells and the presence of intraepithelial bright cells. Nevertheless, they remarked that in the so-called lentiginous pattern of in situ mucosal melanomas, the cytological atypias can be minimal and most architectural criteria can’t be found under RCM, giving more significance to the density of dendritic and/or atypical cells in the basal layer. 

Maher et al. studied on RCM a case series of 8 patients with atypical pigmented lesions of the lip, inclusive of some suspicious for melanoma or melanoma recurrence [32]. Three cases were histopathologically confirmed for in situ melanoma. They focused their attention on the presence of dendritic cells at the epithelial-connective tissue junction, as mystifying element for lip melanoma diagnosis. When dendritic cells were few and located around the connective tissue papillae, they cannot be always considered as malignant melanocytes, as reported by Debarbieux et al. [31]. In these cases, as with all situations, other RCM aspects, have to be considered to confirm a diagnosis of malignancy. The presence of pigment incontinence with melanophages in the superficial stroma can be another confusing factor, as this may appear as bright, large cells on RCM and, therefore, cannot be so easy to distinguish from atypical melanocytes. 

Uribe et al. conducted a retrospective observational study, including six histopathologically proven cases of cutaneous and mucosal lip melanoma, to recognize features useful in the differential diagnosis between benign and malignant pigmented macules of the lip with dermoscopy and RCM [30]. RCM showed a higher frequency of epidermal disarray, pagetoid infiltration of dendritic and/or round cells, a nonspecific architectural pattern at the epithelial connective tissue junction (ECTJ), non-homogenously distributed papillae, continuous (lentiginous) proliferation of cells with marked atypia at the ECTJ (especially in interpapillary spaces), a higher number of dendritic cells and atypical round cells at the ECTJ in melanoma. Based on their observations, Uribe et al. proposed an RCM Lip Score for diagnosing pigmented lip lesions. Using this score, RCM correctly recognized all melanomas as malignant and diagnosed 88% of the melanotic macules as benign, having a sensitivity of 100% and specificity of 88% for melanoma diagnosis if the score was 4 or greater. However, according to the authors themselves, the Lip Score need to be validated in a larger independent study cohort. 

From these studies, mucosal melanoma seems to be characterized by these major features: presence of pagetoid hyper-reflective large cells in the epithelium (mainly roundish or dendritic), high density of basal hyper-reflective large dendritic and round cells and loss of normal architecture of chorion papillae. Data on amelanotic and hypomelanotic mucosal melanoma are lacking; in our experience, atypical cells are still well visible although they are less hyper-reflective and lose reflectance moving from the surface to the inner part of the tumor (Figure 1). 

It should be noted that RCM can also be useful in case of large pigmented mucosal lesions to target initial biopsy sampling, and to perform non-invasive monitoring of foci of melanocytic hyperplasia [33]. 

**Figure 2:**
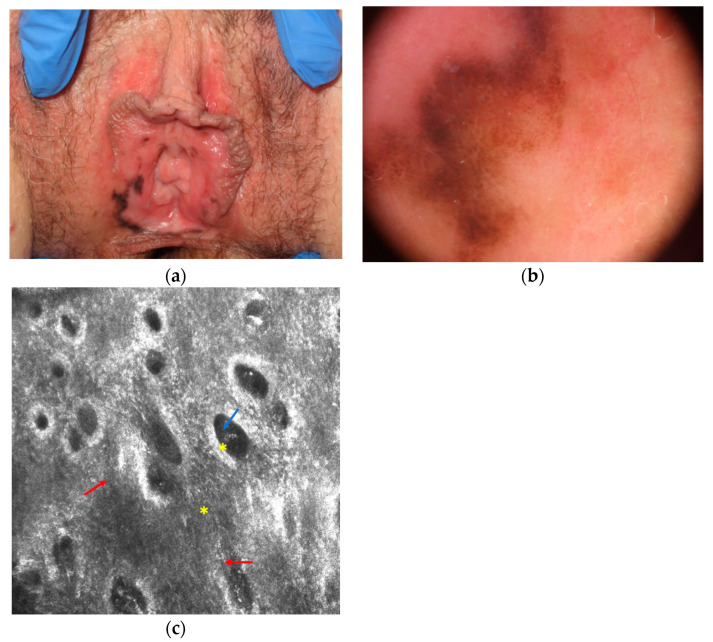
Melanotic macule that is difficult to differentiate from melanoma at clinical (**a**), dermoscopical (**b**) and reflectance confocal microscopy (**c**) examination. Reflectance confocal microscopy (**c**) at the dermal-epidermal junction level shows atypical dendritic cells in the basal layer of the epithelium (red arrows) and normal pigmented epithelial cells (blue arrow) around chorion papillae (yellow asterisk; images acquired with VivaScope 3000, Caliber, New York, NY, USA).

The main clinical, dermoscopical and RCM differential diagnosis of mucosal melanoma is melanotic macule (or melanosis) that is the most common cause of mucosal pigmentation and appear as brown to greyish often large macule with possible multifocal distribution (Figure 2) [5]. Under dermoscopy it shows a parallel, circle and less frequently structureless, reticular-like, and globular pattern [19,20,21]. Although RCM shows hyperpigmented epithelial cells in melanosis without atypical cells, the differential diagnosis could be sometimes difficult due to the possible presence of dendritic bright cells in the basal layer of the epithelium of melanoses, indicating a slight increase in melanocytes or Langerhans cells (Figure 2). However, in melanoma dendritic cells are usually more numerous, larger in size, with shorter and thicker dendrites; and they are located around nonedged and irregular papillae [5,31]. 

We summarized the most important dermoscopic and RCM features of mucosal melanoma in Table 3. Despite dermoscopy and RCM are two useful techniques in detecting mucosal melanoma, in all doubtful cases surgical excision is still mandatory.

## 5. Conclusions

Mucosal melanoma is a rare but often deadly disease, because of the frequent late diagnosis. Dermoscopy and RCM can be two valid technologies for an early and non-invasive diagnosis of mucosal melanoma. Non-invasiveness is very important because of the anatomical district involved, where a biopsy and an excision could be detrimental. The current limitation of these techniques is the absence of standardized and shared diagnostic criteria, due to the rarity of mucosal melanoma and the consequent small number of mucosal melanomas included in imaging studies. Although diagnostic algorithms have been proposed for both methods, all of them need validation on larger studies. In our opinion, the real goal will be to identify imaging criteria that allow an early diagnosis of mucosal melanoma to increase patient survival.

## Figures and Tables

**Table 1:** Dermoscopic features of the mucosal melanomas reported in the literature.

First Author and Reference	Site	Number of Patients	Dermoscopic Features
Hajar-Serviansky [9]	Lip	1	Multicomponent pattern, multiple colors, blue-white veil
Lin [19]	Lip, vulva, anus	7	Multicomponent pattern, homogeneous pattern, blue-white veil (6/7), irregular dots or globules (4/7), regression structure (3/7), irregular vessels (3/7), blotches (2/7)
Ronger-Savle [20]	Vulva	5	Multicomponent pattern (3/5), irregular pattern (2/5), blue-white veil (4/5), irregular dots and globules (4/5), atypical vascular pattern (2/5)
Blum [21]	Lip, vulva, glans, OAA	11	Structureless zones, multiple colors (blue, white, grey), multicomponent pattern (7/11), lines (6/11), dots (4/11), circles (1/11)
Vaccari [22]	Vulva	14	Structureless areas (12/14), grey areas (11/14), irregular black-brown dots (10/14), blu-white structures (10/14)
Blum [23]	Vulva	1	Structureless white area, polymorphous vessels
Rogers [24]	Vulva	1	Structureless areas, blue-pink pigmentation
Kaminska-Winciorek [25]	Oral mucosa	1	Multicomponent pattern, blue-white veil
Matsushita [26]	Lip	1	Irregular diffuse pigmentation with pseudo-network, regression structures, blue-white veil
Simonescu [27]	Lip	1	Polymorphous vascular pattern, blu-white veil, brown globules (nodular area); reticular pattern, homogeneous blue area (macular area)
De Giorgi [28]	Vulva	1	Blue-grey area, whitish veil
Virgili [29]	Vulva	1	Whitish-grey area, irregular globules, irregular vessels
Uribe [30]	Lip	6	Structureless pattern (5/6), asymmetry (5/6) multicomponent pattern (4/6), multiple colors (2/6)

OAA: Other Anogenital Area, E: Epithelium, ECTJ: Epithelial Connective Tissue Junction, CT: Connettive Tis.

**Table 2:** Reflectance confocal microscopy features of the mucosal melanomas reported in the literature.

First Author and Reference	Site	Number of Patients	Reflectance Confocal Microscopy Features
Cinotti [15]	Vulva	2	E: disarranged pattern; roundish, spindle or dendritic pagetoid cells. ECTJ: disarranged papillae; atypical cells in sheet-like structures
Debarbieux [31]	Lip, vulva	10	Fusiform and roundish basal dendritic cells, fusiform and roundish intraepithelial dendritic cells (9/10), sheets of atypical cells (4/10), nests of melanocytes (4/10), pearl-necklace appearance around papillae (2/10), foci of irregular bright thickening of the basal layer (1/10)
Maher [32]	Lip	3	E: typical and atypical honeycomb pattern, cobblestone pattern (2/3); small bright round cells (1/3) and dendritic pagetoid cells (1/3); epidermal disarray (1/3) ECTJ: bright round (2/3) and dendritic cells; nonedged papillae CT: broadened reticulated fibers; small and plump bright cells (1/3)
Uribe [30]	Lip	6	E: atypical architectural pattern; pagetoid infiltration by dendritic (3/6), round (1/6) or both (2/6) cells ECTJ: trabecular or draped pattern (3/6), nonspecific pattern (3/6), nonhomogeneously distributed papillae (5/6), nonedged papillae, continuous proliferation of atypical bright cells (5/6) CT: plump bright cells within the papillae
Theillac [33]	Vulva	1	E: disarranged pattern; roundish, spindle or dendritic pagetoid cells. ECTJ: disarranged papillae; atypical cells in sheet-like structures
Perrot [34]	Cervix (prolapsed)	1	E: pagetoid infiltration by large hyperreflective and polymorphous cells with hyporeflective nucleus ECTJ: same cells

OAA: Other Anogenital Area, E: Epithelium, ECTJ: Epithelial Connective Tissue Junction, CT: Connettive Tis.

**Table 3:** Main dermoscopic and RCM features of mucosal melanoma.

Dermoscopy	RCM
Structureless areas Blue-white veil Multicomponent pattern Multiple colors Irregular vessels	Pagetoid infiltration of hyperreflective and polymorphous cells Atypical pattern of the epithelium Disarranged papillae

## Data Availability

No new data were created or analyzed in this study. Data sharing is not applicable to this article.

## References

[1] Cinotti E., Chevallier J., Labeille B., Cambazard F., Thomas L., Balme B., Leccia M.T., D’Incan M., Vercherin P., Douchet C. (2017). Mucosal melanoma: Clinical, histological andc-kitgene mutational profile of 86 French cases. J. Eur. Acad. Dermatol. Venereol..

[2] Mihajlovic M., Vlajkovic S., Jovanovic P., Stefanovic V. (2012). Primary mucosal melanomas: A comprehensive review. Int. J. Clin. Exp. Pathol..

[3] De Piano E., Cinotti E., Tognetti L., Rubegni P. (2018). Commentary on ‘Oral melanoma and other pigmentations: When to biopsy?’. J. Eur. Acad. Dermatol. Venereol..

[4] González-García R., Naval-Gías L., Martos P.L., Nam-Cha S.H., Rodríguez-Campo F.J., Muñoz-Guerra M.F., Sastre-Pérez J. (2005). Melanoma of the oral mucosa. Clinical cases and review of the literature. Med. Oral Patol. Oral Cir. Bucal.

[5] Cinotti E., Couzan C., Perrot J.L., Habougit C., Labeille B., Cambazard F., Moscarella E., Kyrgidis A., Argenziano G., Pellacani G. (2015). In vivo confocal microscopic substrate of grey colour in melanosis. J. Eur. Acad. Dermatol. Venereol..

[6] Tanaka N., Mimura M., Kimijima Y., Amagasa T. (2004). Clinical investigation of amelanotic malignant melanoma in the oral region. J. Oral Maxillofac. Surg. Off. J. Am. Assoc. Oral Maxillofac. Surg..

[7] Mikkelsen L.H., Larsen A.-C., Von Buchwald C., Drzewiecki K.T., Prause J.U., Heegaard S. (2016). Mucosal malignant melanoma—A clinical, oncological, pathological and genetic survey. APMIS.

[8] Nassar K.W., Tan A.C. (2020). The mutational landscape of mucosal melanoma. Semin. Cancer Biol..

[9] Hajar-Serviansky T., Gutierrez-Mendoza D., Galvan I.L., Lammoglia-Ordiales L., Mosqueda-Taylor A., de Lourdes Hernandez-Cázares M., Toussaint-Caire S. (2012). A case of oral mucosal melanoma. Clinical and dermoscopic correlation. J. Dermatol. Case Rep..

[10] Cinotti E., Labeille B., Debarbieux S., Carrera C., Lacarrubba F., Witkowski A.M., Moscarella E., Arzberger E., Kittler H., Bahadoran P. (2018). Dermoscopy vs. reflectance confocal microscopy for the diagnosis of lentigo maligna. J. Eur. Acad. Dermatol. Venereol..

[11] Tognetti L., Cevenini G., Moscarella E., Cinotti E., Farnetani F., Mahlvey J., Perrot J.L., Longo C., Pellacani G., Argenziano G. (2018). An integrated clinical-dermoscopic risk scoring system for the differentiation between early melanoma and atypical nevi: The iDScore. J. Eur. Acad. Dermatol. Venereol..

[12] Pellacani G., Guitera P., Longo C., Avramidis M., Seidenari S., Menzies S. (2007). The Impact of In Vivo Reflectance Confocal Microscopy for the Diagnostic Accuracy of Melanoma and Equivocal Melanocytic Lesions. J. Investig. Dermatol..

[13] Cinotti E., Labeille B., Cambazard F., Perrot J.L. (2016). Confocal Microscopy for Special Sites and Special Uses. Dermatol. Clin..

[14] Cinotti E., Labeille B., Cambazard F., Thuret G., Gain P., Perrot J.L. (2015). Reflectance confocal microscopy for mucosal diseases. G. Ital. Dermatol. E Venereol. Organo Uff. Soc. Ital. Dermatol. E Sifilogr..

[15] Cinotti E., Perrot J.L., Labeille B., Adegbidi H., Cambazard F. (2012). Reflectance Confocal Microscopy for the Diagnosis of Vulvar Melanoma and Melanosis: Preliminary Results. Dermatol. Surg..

[16] Cinotti E., Haouas M., Grivet D., Perrot J.L. (2015). In Vivo and Ex Vivo Confocal Microscopy for the Management of a Melanoma of the Eyelid Margin. Dermatol. Surg..

[17] Cinotti E., Singer A., Labeille B., Grivet D., Rubegni P., Douchet C., Cambazard F., Thuret G., Gain P., Perrot J.L. (2017). Handheld In Vivo Reflectance Confocal Microscopy for the Diagnosis of Eyelid Margin and Conjunctival Tumors. JAMA Ophthalmol..

[18] Cinotti E., La Rocca A., Labeille B., Grivet D., Tognetti L., Lambert V., Kaspi M., Nami N., Fimiani M., Perrot J.L. (2018). Dermoscopy for the Diagnosis of Conjunctival Lesions. Dermatol. Clin..

[19] Lin J., Koga H., Takata M., Saida T. (2009). Dermoscopy of pigmented lesions on mucocutaneous junction and mucous membrane. Br. J. Dermatol..

[20] Ronger-Savle S., Julien V., Duru G., Raudrant D., Dalle S., Thomas L. (2011). Features of pigmented vulval lesions on dermoscopy. Br. J. Dermatol..

[21] Blum A. (2011). Dermoscopy of Pigmented Lesions of the Mucosa and the Mucocutaneous Junction: Results of a Multicenter Study by the International Dermoscopy Society (IDS). Arch. Dermatol..

[22] Vaccari S., Barisani A., Salvini C., Pirola S., Preti E.P., Pennacchioli E., Iacobone A.D., Patrizi A., Tosti G. (2020). Thin vulvar melanoma: A challenging diagnosis. Dermoscopic features of a case series. Clin. Exp. Dermatol..

[23] Blum A., Beck-Zoul U., Held L., Haase S. (2016). Dermoscopic appearance of an amelanotic mucosal melanoma. Dermatol. Pract. Concept..

[24] Rogers T., Pulitzer M., Marino M.L., Marghoob A.A., Zivanovic O., Marchetti M.A. (2016). Early diagnosis of genital mucosal melanoma: How good are our dermoscopic criteria?. Dermatol. Pract. Concept..

[25] Kamińska-Winciorek G., Calik J., Wydmański J., Schwartz R., Czajkowski R. (2014). Primary melanoma in rare locations: Clinical and dermatoscopic features. Indian J. Dermatol. Venereol. Leprol..

[26] Matsushita S., Kageshita T., Ishihara T. (2005). Comparison of dermoscopic and histopathological findings in a mucous melanoma of the lip. Br. J. Dermatol..

[27] Simionescu O., Dumitrescu D., Costache M., Blum A. (2008). Dermatoscopy of an invasive melanoma on the upper lip shows possible association with Laugier–Hunziker syndrome. J. Am. Acad. Dermatol..

[28] De Giorgi V., Massi D., Salvini C., Mannone F., Cattaneo A., Carli P. (2005). Thin melanoma of the vulva: A clinical, dermoscopic-pathologic case study. Arch. Dermatol..

[29] Virgili A., Zampino M.R., Corazza M. (2004). Primary Vulvar Melanoma with Satellite Metastasis: Dermoscopic Findings. Dermatology.

[30] Uribe P., Collgros H., Scolyer R.A., Menzies S.W., Guitera P. (2017). In Vivo Reflectance Confocal Microscopy for the Diagnosis of Melanoma and Melanotic Macules of the Lip. JAMA Dermatol..

[31] Debarbieux S., Perrot J.L., Erfan N., Ronger-Savlé S., Labeille B., Cinotti E., Depaepe L., Cardot-Leccia N., Lacour J.P., Thomas L. (2014). Reflectance confocal microscopy of mucosal pigmented macules: A review of 56 cases including 10 macular melanomas. Br. J. Dermatol..

[32] Maher N.G., Solinas A., Scolyer R.A., Guitera P. (2017). In vivo reflectance confocal microscopy for evaluating melanoma of the lip and its differential diagnoses. Oral Surg. Oral Med. Oral Pathol. Oral Radiol..

[33] Theillac C., Cinotti E., Malvehy J., Ronger Savle S., Balme B., Robinson P., Perrot J.L., Douchet C., Biron Schneider A.C., Alos L. (2019). Evaluation of large clinically atypical vulvar pigmentation with RCM: Atypical melanosis or early melanoma?. J. Eur. Acad. Dermatol. Venereol..

[34] Perrot J.L., Labeille B., Richard Coulet E., Cochin S., Biron Schneider A.-C., Rubegni P., Cambazard F., Cinotti E. (2017). Apport de la microscopie confocale par réflectance dans le diagnostic d’un mélanome du col utérin: Premier cas rapporté. Ann. Dermatol. Vénéréologie.

[35] Argenziano G., Soyer H.P., Chimenti S., Talamini R., Corona R., Sera F., Binder M., Cerroni L., De Rosa G., Ferrara G. (2003). Dermoscopy of pigmented skin lesions: Results of a consensus meeting via the Internet. J. Am. Acad. Dermatol..

[36] Hofmann-Wellenhof R. (2013). Special Criteria for Special Locations 2. Dermatol. Clin..

[37] Cinotti E., Campoli M., Pataia G., Ouerdane Y., Thuret G., Gain P., Tognetti L., Perrot J.L., Rubegni P. (2019). How transparent film applied on dermatologic imaging devices in order to prevent infections affects image quality?. Skin Res. Technol..

